# Prevalence of Painful Diabetic Peripheral Neuropathy Among Saudi Patients With Diabetes in Al Ahsa: A Cross-Sectional Study

**DOI:** 10.7759/cureus.49317

**Published:** 2023-11-23

**Authors:** Rasmah S Alharajin, Hessa S Al Moaibed, Fatimah K Al Khalifah

**Affiliations:** 1 Department of University Health Clinic, King Saud Bin Abdulaziz University for Health Sciences, Al Ahsa, SAU; 2 Department of Family Medicine, King Abdulaziz Hospital, Ministry of the National Guard, Al Ahsa, SAU

**Keywords:** al ahsa, saudi arabia, primary healthcare, prevalence, painful peripheral diabetic neuropathy, diabetes mellitus

## Abstract

Background and aim

Diabetic neuropathy is one of the most common long-term complications of diabetes. A frequent presentation of neuropathy is painful diabetic peripheral neuropathy (PDPN), which is associated with morbidity and disability among patients with diabetes. This study aims to estimate the prevalence of PDPN and its associated factors among patients with type 2 diabetes mellitus (T2DM) attending primary healthcare (PHC) in Al Ahsa.

Methodology

A cross-sectional study was conducted on patients with diabetes at National Guard PHC clinics in Al Ahsa, Saudi Arabia. An interview-administered questionnaire was used to collect information about clinicodemographic characteristics, and the Douleur Neuropathique-4 (DN4) questionnaire was used to identify the presence of PDPN.

Results

A total of 342 patients with T2DM were included. The prevalence of PDPN was 29.8%. Patients' ages ranged from 25-70 years, with a mean age of 48.5 ± 12.3 years. The majority were female (118, 52.6%), with obesity detected among 115 (33.6%) individuals. Significant predictors of PDPN included noncompliance to treatment (odds ratio [OR] = 5.9, *P* = 0.001), female gender (OR = 3.5, *P* = 0.001), presence of other comorbidities (OR = 1.7, *P* = 0.001), and diabetes duration exceeding 15 years (OR = 1.5, *P* = 0.001).

Conclusions

This study revealed that PDPN was frequent among patients with diabetes in Al Ahsa, which was at an intermediate frequency relative to reported national and global literature levels. Identifying patients who are at high risk and implementing timely interventions are crucial.

## Introduction

Diabetes mellitus (DM) is prevalent worldwide and affects about 537 million adults, and this number is expected to increase to 643 million by the year 2035 and 783 million by 2045 [1].

Diabetic neuropathy is one of the most common long-term complications of diabetes, which is a debilitating condition that occurs in nearly half of patients with diabetes and is associated with substantial morbidity, including pain, foot ulcers, and lower limb amputation [2-4]. Diabetic neuropathy is a very broad and heterogeneous term encompassing various clinical and subclinical presentations [5]. Painful diabetic peripheral neuropathy (PDPN) is a common presentation of diabetic neuropathy, which could be described as a burning or stabbing pain, numbness, hyperesthesia, or a deep ache with nocturnal exacerbation, and typically affects the feet and lower extremities. However, in some patients, the hands also can be affected [2,6,7]. In addition to causing pain, diabetic neuropathy has significant adverse effects on the quality of life, daily activities, and sleep of patients. It can also lead to psychological issues such as anxiety and depression, as well as pose a substantial medical burden [5,8,9].

According to the literature, several factors have been linked to PDPN risk, including demographic characteristics, diabetic complications, lifestyle, biochemical indicators, and genetic factors [10]. In Saudi Arabia, PDPN is significantly common; the overall prevalence across the country was reported in 65.3% of patients with diabetes in a previous study [11]. Of three other studies conducted in the primary healthcare (PHC) setting, the prevalence of PDPN varied from 29% to 35% [2,12,13]. Despite the high prevalence, PDPN is frequently underdiagnosed, unreported, and inadequately treated [10,14]. In addition, it was noted in a recent review that the knowledge of the epidemiology of PDPN is compromised by the differences in study populations and diagnostic criteria, which can lead to conflicting findings among studies [14]. In Al Ahsa, Saudi Arabia, the data related to the prevalence of PDPN are lacking.

Therefore, this study aimed to estimate the prevalence of PDPN and its associated factors among patients with type 2 diabetes mellitus (T2DM) attending PHC in Al Ahsa using the Douleur Neuropathique-4 (DN4) questionnaire, which is a valid and reliable screening tool for neuropathic pain [2,15]. The study's results may increase the awareness of PDPN, encourage aggressive screening, and provide medical evidence for early clinical intervention and active prevention.

## Materials and methods

This was a cross-sectional study carried out in PHC clinics affiliated with the National Guard Health Affairs in Al Ahsa, namely, Family Medicine Clinics Center at King Abdulaziz Hospital (KAH) and Family Medicine Clinics Center at Iskan.

Three hundred and forty-two patients were enrolled between July and October 2023. Patients were included if they were between the ages of 25 and 70 and had a confirmed diagnosis of T2DM. Patients with type 1 DM, gestational diabetes, neurologic disorders, neuropathic pain of nondiabetic origin, history of nerve root compression, malignancy, or alcoholism were excluded.

The sample size was based on 65.3% PDPN prevalence [11], 80% power, and 5% significance. We adopted the random cluster sampling technique; patients were sampled randomly and equally sampled (*n* = 171) from each of the two centers.

After obtaining written informed consent, the data were collected using an interview-administered questionnaire consisting of two sections. The first section constituted the clinicodemographic data. The second section is the DN4 questionnaire, a tool used to discover the symptoms of PDPN. It is a valid and reliable screening tool with a sensitivity of 80% and specificity of 92% [15]. The original version was validated by Bouhassira et al. [16], and the Arabic version was validated by Terkawi et al. [17].

DN4 includes 10 items: the first seven items of the DN4 are based on an analysis of the patient's sensory description of their pain, and the last three items are based on a physical evaluation of sensory function. Each question had only two options: *yes* or *no*. Responses to *Yes* received one point, while responses to *No* received none. The total score can vary from 0 to 10, and a value of 4 or higher is considered the diagnostic threshold for neuropathic pain.

This study protocol was approved by the ethical committee of the Institutional Review Board at King Abdullah International Medical Research Center (KAIMRC). Participants were assured about the confidentiality and the purpose of using the obtained data.

After data were extracted, they were revised, coded, and fed to statistical software IBM SPSS Version 22 (SPSS Inc., Chicago, IL). All statistical analyses were done using two-tailed tests. A *P*-value less than 0.05 was statistically significant. Descriptive analysis based on frequency and percent distribution was done for all. Additionally, patients' pain characteristics and associated symptoms, besides physical examination findings, were tabulated, and the overall prevalence of PDPN was graphed. Cross-tabulation was used to assess factors associated with PDPN using Person's chi-square test and the exact probability test for small frequency distributions. Multiple stepwise logistic regression model was used to determine the most significant predictors of having PDPN among study patients (adjusted relations).

## Results

A total of 342 patients with diabetes were included. Patients' ages ranged from 25 to 70 years, with a mean age of 48.5 ± 12.3 years. Exactly 180 (52.6%) of the patients were females, with obesity detected in 115 (33.6%), while 158 (46.2%) were overweight. Approximately 75 (21.9%) were smokers (Table 1).

**Table 1:** Personal characteristics of patients with diabetes in Al Ahsa, Saudi Arabia.

Personal data	Number, *n*	%
Age in years		
25-35	27	7.9
36-45	55	16.1
46-55	113	33
>55	147	43
Gender		
Male	162	47.4
Female	180	52.6
BMI		
Normal	69	20.2
Overweight	158	46.2
Obesity	115	33.6
Current smoking		
Yes	75	21.9
No	267	78.1

A total of 115 (33.6%) patients had diabetes for five to 10 years, 93 (27.2%) were diabetic for 11 to 15 years, and 70 (20.5%) were diabetic for less than five years. A total of 179 (52.3%) patients were on oral hypoglycemics, 39 (11.4%) used insulin injections, and 116 (33.9%) used both of them, while only 8 (2.3%) needed lifestyle modification. About 234 (68.4%) were compliant with treatment. As for diabetes control, 154 (45%) had controlled blood glucose levels (HbA1c < 7%), while 113 (33%) had high levels and 75 (21.9%) had very high HbA1c. Considering comorbidities, 24 (7%) were hypertensive, 69 (20.2%) had dyslipidemia, and the majority of them (200, 58.5%) had both (Table 2).

**Table 2:** Bioclinical data of diabetes among study patients in Al Ahsa, Saudi Arabia. HbA1c, glycated hemoglobin; DM, diabetes mellitus

Clinical data	Number, *n*	%
Duration of DM in years		
<5	70	20.5
5-10	115	33.6
11-15	93	27.2
>15	64	18.7
Medications for diabetes		
Oral hypoglycemic	179	52.3
Insulin	39	11.4
Both	116	33.9
Lifestyle modification	8	2.3
Compliance with treatment		
Yes	234	68.4
No	108	31.6
HbA1c		
Controlled (<7.0)	154	45
High (7.0-9.0)	113	33
Very high (>9.0)	75	21.9
Other comorbidities		
None	49	14.3
Hypertension	24	7
Dyslipidemia	69	20.2
Both	200	58.5

As for pain characteristics, 135 (39.5%) of the patients had burning pain, 53 (15.5%) had electric shock pain, and 28 (8.2%) had painful cold extremities. As for associated symptoms, 181 (52.9%) had numbness with pain, 141 (41.3%) had pins and needles sensation, 90 (26.3%) had tingling, and 77 (22.5%) had itching. Physical examination showed that 29 (8.5%) experienced hypoesthesia to touch, 24 (7%) reported that pain was caused or increased by brushing on a painful area, and 21 (6.1%) had hypoesthesia to pinprick (Table 3).

**Table 3:** Diabetic peripheral neuropathy pain among study patients in Al Ahsa, Saudi Arabia.

Domain	Items	Number, *n*	%
Patient's sensory description of their pain	Pain characteristics		
	Burning	135	39.5
	Painful cold	28	8.2
	Electric shocks	53	15.5
	Pain-associated symptoms		
	Tingling	90	26.3
	Pins and needles	141	41.3
	Numbness	181	52.9
	Itching	77	22.5
Physical evaluation of sensory function	Physical examination findings		
	Hypoesthesia to touch	29	8.5
	Hypoesthesia to pinprick	21	6.1
	In the painful area, can the pain be caused or increased by brushing?	24	7

**Figure 1:**
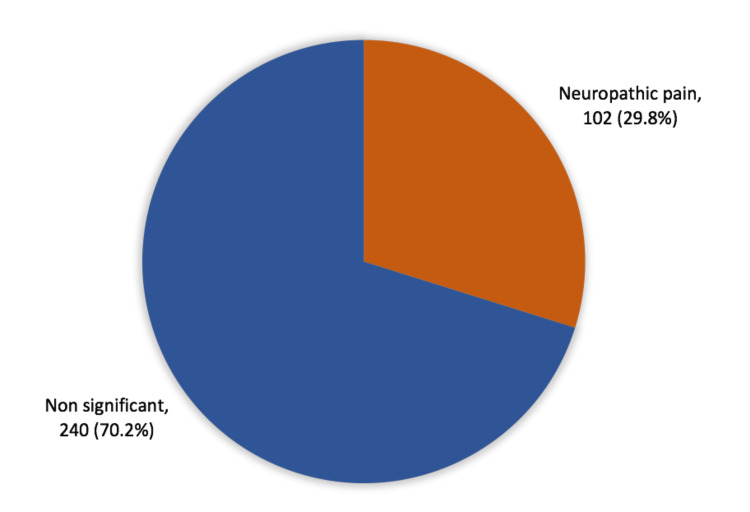
Prevalence of painful diabetic peripheral neuropathy among Saudi patients with diabetes in Al Ahsa, Saudi Arabia.

A total of 102 (29.8%) of the study patients had neuropathic pain, while the majority of them (240, 70.2%) had nonsignificant neuropathic pain (Figure 1).

Exactly 41.5% (61) of patients aged more than 55 years had PDPN compared to 14.8% (4) of those aged less than 35 years, with recorded statistical significance (*P* = 0.001). Also, PDPN was detected among 41.1% (74) of female patients compared to 17.3% (28) of males (*P* = 0.001). PDPN was detected in 38.3% (44) of obese patients compared to 5.8% (4) of those with a normal BMI (*P* = 0.001). Likewise, 45.5% (91) of patients with diabetes who also had hypertension and dyslipidemia experienced PDPN compared to 8.2% (4) of those with no comorbidities (*P* = 0.001). PDPN was also detected among 33.3% (89) of nonsmokers compared to 17.3% (13) of smokers (*P* = 0.007). A total of 48.4% (31) of patients with diabetes for more than 15 years had PDPN, in comparison to 5.7% (4) of those with DM for less than five years (*P* = 0.001). Additionally, 69.2% (27) of patients on insulin had PDPN compared to none of the others who needed lifestyle modification (*P* = 0.001). PDPN was detected among 73.1% (79) of noncompliant patients to treatment versus 9.8% (23) of others who were compliant (*P* = 0.001). Also, 90.7% (68) of patients with poor diabetic control had PDPN compared to 1.9% (3) of others with controlled blood glucose levels (*P* = 0.001) (Table 4).

**Table 4:** Factors associated with painful diabetic peripheral neuropathy among study patients. P: Pearson X2 test. ^$^Exact probability test. ^*^P < 0.05 (significant). BMI, body mass index; HbA1c, glycated hemoglobin; DM, diabetes mellitus

Factors	Neuropathic pain				*P*-value
	Neuropathic pain		Nonsignificant		
	Number, *n*	%	Number, *n*	%	
Age in years					0.001*^$^
25-35	4	14.8	23	85.2	
36-45	8	14.5	47	85.5	
46-55	29	25.7	84	74.3	
>55	61	41.5	86	58.5	
Gender					0.001*
Male	28	17.3	134	82.7	
Female	74	41.1	106	58.9	
BMI					0.001*
Normal	4	5.8	65	94.2	
Overweight	54	34.2	104	65.8	
Obesity	44	38.3	71	61.7	
Other comorbidities					0.001*^$^
None	4	8.2	45	91.8	
Hypertension	2	8.3	22	91.7	
Dyslipidemia	5	7.2	64	92.8	
Both	91	45.5	109	54.5	
Currently smoking					0.007*
Yes	13	17.3	62	82.7	
No	89	33.3	178	66.7	
Duration of DM in years					0.001*
<5	4	5.7	66	94.3	
5-10	30	26.1	85	73.9	
11-15	37	39.8	56	60.2	
>15	31	48.4	33	51.6	
Medications for diabetes					0.001*^$^
Oral hypoglycemic	17	9.5	162	90.5	
Insulin	27	69.2	12	30.8	
Both	58	50	58	50	
Lifestyle modification	0	0.0	8	100	
Compliance to treatment					0.001*
Yes	23	9.8	211	90.2	
No	79	73.1	29	26.9	
HbA1c					0.001*
Controlled (<7.0)	3	1.9	151	98.1	
High (7.0-9.0)	31	27.4	82	72.6	
Very high (>9.0)	68	90.7	7	9.3	

As illustrated in Table 5, among all included risk factors, those shown in the table were the most significant predictors of having PDPN among patients with diabetes. Uncontrolled diabetes increased the risk for PDPN by about 12 times (OR = 11.7), noncompliance to treatment increased the risk by about six times (OR = 5.9), female gender increased the risk by about four times (OR = 3.5), having other comorbidities nearly doubled the risk for PDPN (OR = 1.7), and longer duration of diabetes increased the risk for PDPN by about 50% (OR = 1.5).

**Table 5:** Multiple stepwise logistic regression model for predictors of painful diabetic peripheral neuropathy among study patients. ^*^P < 0.05 (significant). B, regression coefficient; OR_A_, adjusted odds ratio; CI, confidence interval; DM, diabetes mellitus

Factors	B	SE	Sig.	OR_A_	95% CI	
					Lower	Upper
Female vs. male	1.3	0.4	0.003*	3.5	1.5	8.1
Duration of DM	0.4	0.2	0.049*	1.5	1.0	2.3
Noncompliance with treatment	1.8	0.4	0.001*	5.9	2.6	13.2
Comorbidities	0.5	0.2	0.036*	1.7	1.0	2.6
Uncontrolled DM	2.5	0.4	0.001*	11.7	5.5	24.9

## Discussion

This study aimed to estimate the prevalence of PDPN among patients with T2DM attending PHC clinics in Al Ahsa, Saudi Arabia. In this setting, 29.3% of patients with diabetes met the criteria for PDPN (DN4 score ≥ 4). In more detail, more than one-third of the patients had burning pain, but electric shock pain and painful cold extremities were infrequent. When considering associated symptoms, approximately half of the patients experienced numbness with pain, a lower percentage reported pins and needles sensation, and only one-fourth had tingling. Additionally, about one-fifth of them reported itching. Physical examination showed that hypoesthesia to touch, hypoesthesia to pinprick, and increased pain by brushing on a painful area were infrequent among study patients. Of all factors considered, female gender, long duration of diabetes, glycemic control, noncompliance to treatment, and comorbid conditions emerged as the most significant predictors for developing PDPN.

The previous studies on the prevalence of PDPN have reported different prevalence results. A higher prevalence was reported by Li et al. [10] as 57.2% of patients with diabetes experienced PDPN. Also, this study's findings were lower than that estimated in a study conducted in Saudi Arabia where PDPN was reported as 65.3% [11], in Libya at 42.2% [18], in the United Kingdom at 34% [19], and in South Africa at 30.3% [20]. On the other hand, Abdissa [21] documented that the prevalence of PDPN was 14.5% among patients with diabetes, which was much lower than the estimated in this study patients. Also, other studies revealed lower incidence for PDPN such as 15.3% in Morocco [22], 16% in Turkey [23], 16.7% in Brazil [24], 14% in Belgium [25], and 22.1% in Japan [26]. Prevalence rates vary widely within the country and among diverse countries due to variations in the patient populations studied and the different criteria used to define PDPN.

This study revealed that less than half of the patients had controlled blood glucose levels, as only two-thirds of them were compliant with the prescribed treatment. This strong association of PDPN with lack of tight glycemic control and incompliance with treatment was consistent with the findings of other studies [12,23,27]. Our results, in agreement with previous research findings [2,17,23], indicated that female gender and a longer duration of DM were significantly associated with higher odds of PDPN. The majority of the study participants were overweight/obese and had other comorbidities, including hypertension and dyslipidemia. These findings have been confirmed by other studies [28,29]. We observed no significant association between PDPN and smoking status, unlike the previous study reported by Aslam et al. [30].

Study limitations

There are a few limitations of this study that need to be addressed. First, the sample may not be fully representative of all patients with diabetes in Al Ahsa, as it was conducted in a National Guard PHC setting. Generalizations to the total population of patients with diabetes in Al Ahsa should, therefore, be undertaken with caution. Second, using a cross-sectional design prevents determining causality between the established risk factors and painful peripheral diabetic neuropathy, so more study is needed to follow patients over time and observe changes in prevalence or associated factors. Third, additional studies are needed to explore the impact of the condition on patients' quality of life, daily activities, or the effectiveness of various treatments. Addressing these limitations in future research can contribute to a more comprehensive understanding of PDPN and its implications for patients with diabetes.

## Conclusions

In conclusion, this study revealed that PDPN was frequent among patients with diabetes in Al Ahsa, at an intermediate frequency relative to reported national and global literature levels. High variability may be attributed to the method of diagnosing PDPN and patient characteristics. Females with a long duration of DM, poor compliance to treatment, or the presence of other comorbidities were the main factors contributing to the development of PDPN. Identifying patients who are at high risk and implementing timely interventions are crucial. It is important to prioritize screening for patients with PDPN who are older, have longer T2DM duration, have poor treatment compliance, and have comorbidities. 

We can conclude that approximately one-third of patients with diabetes in Al Ahsa, Saudi Arabia, have PDPN, indicating a significant presence of neuropathic pain in this population.

Risk factors for developing PDPN include being female, having a longer duration of diabetes, noncompliance with treatment, and the presence of comorbidities. These factors should be considered when diagnosing and treating patients with diabetes experiencing neuropathic pain.

Effective management of diabetes is crucial, as there is a strong association between PDPN and uncontrolled diabetes. Patients with poor glycemic control are more likely to experience neuropathic pain. Noncompliance with prescribed treatment is also a significant predictor of PDPN.

Screening programs are necessary to identify patients with diabetes at higher risk of developing PDPN. Early preventive measures can be initiated by physicians to reduce the prevalence of the disease. Appropriate therapies can minimize neuropathic pain for individuals with specific risk factors such as long-term diabetes, noncompliance with treatment, and comorbidities.

Increasing awareness about pain relief for patients with PDPN can improve their quality of life and reduce the financial burden associated with the disease.

## References

[1] Karuranga S, Saeedi P, Malanda B (2019). IDF Diabetic Atlas [Internet]. https://diabetesatlas.org/upload/resources/material/20200302_133351_IDFATLAS9e-final-web.pdf.

[2] AlSufyani MH, Alzahrani AM, Allah AA, Abdullah RI, Alzhrani SH, Alsaab AA (2020). Prevalence of painful diabetic peripheral neuropathy and its impact on quality of life among diabetic patients in Western region, Saudi Arabia. J Family Med Prim Care.

[3] Aring AM, Jones DE, Falko JM (20052023). Evaluation and prevention of diabetic neuropathy. Am Fam Physician [Internet.

[4] Hicks CW, Selvin E (2019). Epidemiology of peripheral neuropathy and lower extremity disease in diabetes. Curr Diab Rep.

[5] Kaur S, Pandhi P, Dutta P (2011). Painful diabetic neuropathy: an update. Ann Neurosci.

[6] Zaheer A, Zaheer F, Saeed H, Tahir Z, Tahir MW (2021). A review of Alternative treatment options in diabetic polyneuropathy. Cureus.

[7] Huizinga MM, Peltier A (2007). Painful diabetic neuropathy: a management-centered review. Clin Diabetes.

[8] Schreiber AK, Nones CF, Reis RC, Chichorro JG, Cunha JM (2015). Diabetic neuropathic pain: physiopathology and treatment. World J Diabetes.

[9] Gore M, Brandenburg NA, Dukes E, Hoffman DL, Tai KS, Stacey B (2005). Pain severity in diabetic peripheral neuropathy is associated with patient functioning, symptom levels of anxiety and depression, and sleep. J Pain Symptom Manage.

[10] Li C, Wang W, Ji Q (2023). Prevalence of painful diabetic peripheral neuropathy in type 2 diabetes mellitus and diabetic peripheral neuropathy: a nationwide cross-sectional study in mainland China. Diabetes Res Clin Pract.

[11] Halawa MR, Karawagh A, Zeidan A, Mahmoud AE, Sakr M, Hegazy A (2010). Prevalence of painful diabetic peripheral neuropathy among patients suffering from diabetes mellitus in Saudi Arabia. Curr Med Res Opin.

[12] Sendi RA, Mahrus AM, Saeed RM, Mohammed MA, Al-Dubai SA (2020). Diabetic peripheral neuropathy among Saudi diabetic patients: a multicenter cross-sectional study at primary health care setting. J Family Med Prim Care.

[13] Algeffari MA (2018). Painful diabetic peripheral neuropathy among Saudi diabetic patients is common but under-recognized: Multicenter cross-sectional study at primary health care setting. J Family Community Med.

[14] Davies M, Brophy S, Williams R, Taylor A (2006). The prevalence, severity, and impact of painful diabetic peripheral neuropathy in type 2 diabetes. Diabetes Care.

[15] Spallone V, Morganti R, D'Amato C, Greco C, Cacciotti L, Marfia GA (2012). Validation of DN4 as a screening tool for neuropathic pain in painful diabetic polyneuropathy. Diabet Med.

[16] Bouhassira D, Attal N, Alchaar H (2005). Comparison of pain syndromes associated with nervous or somatic lesions and development of a new neuropathic pain diagnostic questionnaire (DN4). Pain.

[17] Terkawi AS, Abolkhair A, Didier B (2017). Development and validation of Arabic version of the douleur neuropathique 4 questionnaire. Saudi J Anaesth.

[18] Garoushi S, Johnson MI, Tashani OA (2019). A cross-sectional study to estimate the point prevalence of painful diabetic neuropathy in Eastern Libya. BMC Public Health.

[19] Abbott CA, Malik RA, van Ross ER, Kulkarni J, Boulton AJ (2011). Prevalence and characteristics of painful diabetic neuropathy in a large community-based diabetic population in the U.K. Diabetes Care.

[20] Jacovides A, Bogoshi M, Distiller LA (2014). An epidemiological study to assess the prevalence of diabetic peripheral neuropathic pain among adults with diabetes attending private and institutional outpatient clinics in South Africa. J Int Med Res.

[21] Abdissa D (2020). Prevalence and associated factors of painful diabetic peripheral neuropathy among diabetic patients on follow up at Jimma University Medical Center. J Diabetes Metab Disord.

[22] Chahbi Z, Lahmar B, Hadri SE (2018). The prevalence of painful diabetic neuropathy in 300 Moroccan diabetics. Pan Afr Med J.

[23] Erbas T, Ertas M, Yucel A, Keskinaslan A, Senocak M (2011). Prevalence of peripheral neuropathy and painful peripheral neuropathy in Turkish diabetic patients. J Clin Neurophysiol.

[24] Cortez J, Reis CCCD, Cardoso Y, Onofre A, Piovezan AP (2014). Prevalence of neuropathic pain and associated factors in diabetes mellitus type 2 patients seen in outpatient setting. Revista DOR.

[25] Van Acker K, Bouhassira D, De Bacquer D (2009). Prevalence and impact on quality of life of peripheral neuropathy with or without neuropathic pain in type 1 and type 2 diabetic patients attending hospital outpatients clinics. Diabetes Metab.

[26] Tsuji M, Yasuda T, Kaneto H (2013). Painful diabetic neuropathy in Japanese diabetic patients is common but underrecognized. Pain Res Treat.

[27] Young E, Nwatu CB, Ekenze O (2019). Prevalence of painful diabetic peripheral neuropathy among patients with diabetes attending a tertiary outpatient diabetes clinic in Nigeria. Journal of Advances in Medicine and Medical Research.

[28] Spallone V, Morganti R, D'Amato C, Cacciotti L, Fedele T, Maiello MR, Marfia G (2011). Clinical correlates of painful diabetic neuropathy and relationship of neuropathic pain with sensorimotor and autonomic nerve function. Eur J Pain.

[29] Barbosa M, Saavedra A, Oliveira S (2019). Prevalence and determinants of painful and painless neuropathy in type 1 diabetes mellitus. Front Endocrinol (Lausanne).

[30] Aslam A, Singh J, Rajbhandari S (2015). Prevalence of painful diabetic neuropathy using the self-completed Leeds assessment of neuropathic symptoms and signs questionnaire in a population with diabetes. Can J Diab.

